# British Periodical Press

**Published:** 1828-04-01

**Authors:** 


### BRITISH PERIODICAL PRESS.

### 1. ED. MED. & SURG. JOURNAL.

The race of medical periodicals cannot boast of high pedigree. But what they want in genealogy they make up in fecundity. There was a time, ('tis sixty years since) when our grandfathers brought forth much less frequently than our grandmothers—and when the sun paid a visit to all the signs in the zodiac, during each term of literary pregnancy! It was in that peaceful period the dynasty in question commenced, under the modest appellation of "MEDICAL COMMENTATORS" —for critics were not then known in medical literature. The ostensible object of our forefathers was precisely what is now alleged by their numerous progeny. "A scheme, better calculated for saving time in reading, and expense in purchasing books, is a concise view of the books themselves."—*Introd. to vol.* i. *Jan.* 1773. Thus we see that our Caledonian ancestors had an eye to economy, even in those days.*

The dynasty of COMMENTATORS lasted 20 years and upwards, when, without any very apparent cause, it changed its title to ANNALISTS, (ANNALS of MEDICINE) but still pursued its slow annual phases, returning with Christmas, or the new year, to add to the enjoyments of those festive epochs. The ANNALS continued from 1796 to 1805, when an impulse was evinced, that might well be deemed prophetic of what is now taking place, after a lapse of 20 years. Again the family name was changed—the publication was made QUARTERLY, instead of ANNUAL, and the Journal at the head of this article started into existence.† It appears that, even then, it was customary to declaim against "the multitude of similar publications." Where are the records of this multitude? The following sentence will explain. "All such periodical works, from the very nature and origin of them, have their periods of vigour and decay. They flourish, and they fade. They terminate, abruptly; or, after reaching a respectable old age, they are suspended. New ones arise. Their powers are rapidly developed, &c." And, we may add, they follow the fate of their predecessors, sooner or later! Gibbon tells us that, in the Byzantine empire, although the grave was dug at the very foot of the throne, the LATTER never wanted a tenant, however soon he was destined to descend into the gloomy habitation below! The dynasty of the Duncans has had a long and honorable career. The three series, spread over a space of half a century, have maintained a more uniform character, dignity, and respectability, than any work of the kind, in this or any other country. The cause of this uniformity was, doubtless, owing to the government of the journal being patriarchal, and, therefore, not subjected to perpetual vicissitudes in its editorial management. The innumerable contributions to this work varied with the contributors—and although many able and erudite reviews are scattered through its volumes, we cannot say that the work, as a whole, has evinced any very powerful talent, or exuberant principle of vitality. Its original conductors have, we believe resigned—and how far its present Editors may enhance, maintain, or diminish the fame of this father of British periodicals, in an age when all the energies of the human mind are called forth, it is not for us to predicate.

* The Edinburgh Journal has sadly diverged from its original purpose. No one now expects any thing like an account of the medical literature of the day in the review department of that work.

† Let those who startle at changes in the mode of publication, look back at the history of periodicals, and they will find changes enough. They will find that, in almost every instance, the change has been for the better, when the periods of publication were shortened.

### No. 94.—JAN. 1st, 1828.

This Number of our Edinburgh cotemporary contains 13 original papers, occupying 118 pages of the work, and evincing various degrees of merit. The first on the list is that which we shall notice in the present fasciculus of our Journal.

*Treatment of Caries.* By Dr. J. J. NICOL.

After some desultory remarks on the diseases of bone generally, Dr. N. comes to the pith of the communication—the TREATMENT, by the application of lunar caustic to the bone or its periosteum, aided or not by the internal use of sarsaparilla, and general constitutional remedies. Dr. N. modestly premises, that he offers these cases to the profession, "without the least pretensions to novelty, in so far as regards the *remedies* employed." What, then, it may be asked, was the object of publication? "The success of their application alone has a claim on its attention." Here the modesty is not quite so striking as in the preceding sentence. If the remedies are not novel, but only the success of their application remarkable, then the operator has the credit. Be this as it may, a single case will illustrate the mode of treatment pursued by our author, as well as a whole sheet of didactic precepts.

"*Case* 2.—A young gentleman, about 16 years of age, having over-exerted himself in running, was seized with inflammation along the right shin, extending towards the knee. The surgeon who attended leeched and applied evaporating sedative lotions, by which the inflammatory process was in a great measure subdued. Ulceration however followed some time after along the flat upper part of the tibia; and the integuments gave way *in two* places, about the distance of an inch and a half from each other. The discharge became limited and unhealthy; the upper end of the tibia enlarged; the knee excessively pained; and the general health bad. Leeches and evaporating lotions were again applied over the knee without any benefit, and diaphoretics were also freely administered without any relief. The case becoming alarmingly retrograde, and the parents anxious, I was called in. The knee was much swollen, and the leech-bites had become vesicated, presenting the appearance of pemphigus. The whole upper end of the tibia, and for some inches downwards, was evidently enlarged; its anterior tubercle projected unusually, and was soft, but not tender to the touch. The orifices of the ulcerations previously described had enlarged to nearly an inch in diameter, communicating freely with each other, and exposing the periosteum, which was glairy, and discharged a thin sero-gelatinous rather than sero-purulent matter. Hectic had existed for some time, and the strength was much exhausted. The tibia was now pronounced in a state of extensive disease internally, and the treatment completely reversed. An incision with a scalpel was made on the outside of the enlarged tuberosity, and a rod of caustic introduced close to the bone. One single point of the periosteum at each of the ulcerations below was touched in a similar manner. Fomentations of poppy capsule were applied two or three times daily; four ounces of *Decoct. Sarsaparillæ comp.* were given morning, noon, and night; and from half a grain to a grain of opium exhibited two hours after each dose of the decoction.

"In twenty-four hours tranquillity was restored to the limb. In three days the hectic fell off. This plan of treatment was continued, substituting for the opium as many tea-spoonfuls of the following mixture as the stomach could bear without sickness or nausea.

"℞. *Antim. tartarizat. gr.* iv.
*Solv. in aq. cinnam.* ℥viij.
*Tinc. opii,* ʒj. *M.*"

The swelling of the knee disappeared —that in the bone diminished—and two thin circular films (evidently separations from the outer lamina of the tibia) were discharged at the openings. The ulcers then turned healthy, and, in about four months from the time Dr. N. first saw the patient, a cure was effected, and the young gentleman is now a useful *limb* of the profession himself.

Dr. N. makes several judicious observations on individual remedies, employed in these diseases of the bones and their coverings. General blood-letting can only be resorted to in the acute stages, where the inflammation partakes of the gouty or rheumatic character. Instead of antimony, opium, and calomel, and other remedies, formerly much employed, our author now depends greatly on colchicum, with Battley's liq. opii sedativus. These he uses in the proportion of two or three parts of the former to one of the latter—thus, giving to an adult 30 drops, or more, (of the combination) every three or four hours, according to the urgency of the symptoms, continuing the medicine till the stomach or bowels become affected, or copious perspiration is excited.

When, by these means, aided by sarsaparilla, and proper local treatment, the disease has been arrested, but the bone still continues enlarged and the periosteum thickened, then the establishment of a caustic issue becomes necessary. "When the most prominent part can be easily reached, I have invariably made an incision with the point of a scalpel, down to the bone, and not larger than was sufficient to admit a pointed rod of lunar caustic, of the usual size, which was immediately introduced, twirled round two or three times, and instantly withdrawn." A poultice was then applied, till the eschar separated, and a purulent discharge was established. Dr. N. affirms that—"short of the actual excision of the parts, he considers the lunar caustic to be the sheet anchor in the treatment of affections of the bones."

We shall notice the other articles in succession—at least all those which we deem practically useful or theoretically interesting. And we take this opportunity of observing, that our Periscope of Journals will always partake of the character which we have endeavoured to establish for our Analytical Review of Books. We shall not give a bare catalogue—or a meagre skeleton of papers, divested of blood, nerve, and flesh. Of those articles which we do notice, we shall aim at giving a concise, but intelligible view of their import and bearing, not a tantalizing glimpse, conveying no definite ideas to the minds of our readers. Let this be borne in mind.

---

### 2. THE LONDON MEDICAL REPOSITORY.

This journal started on a flood-tide of popularity that promised to carry it to immortality. It was presented to the public as "the Surgeon-Apothecaries' and Apothecaries' Journal and Review," in the year 1814, when a strong excitement and impulse existed throughout that wide and respectable class of medical society, to distinguish itself by science, art, and literature. The journal was conducted by three eminent general practitioners—Messrs. Burrows, Royston, and Thomson—so that every thing conspired to the success of the work. This success was great—but it was astonishingly over-rated. The first number circulated to the extent of 1250, and this point was never afterwards over-stepped. Four years later, (1818,) when the property was sold to Messrs. Underwood, the circulation had dropped to 1000. One of its professed objects was—"to bring within the reach of the country practitioner whatever of novelty arises in theory or practice"—and to "assist in the amelioration of medical literature, by inciting to the practice of composition." In respect to their review department, the editors were inclined to think—"*that* analysis which gives a faithful view of facts to be more useful to the public, than *that* criticism which aims to exalt the critic above the author." Most people are now of the same opinion.

The London Medical Repository has passed through many hands during the last ten years, and consequently taken its hue and intellectual character from its reigning master or masters.* While, originally, the organ of the general practitioners, it strenuously advocated the interests of that body, but never insulted any other class of medical society. The consequence was, that it had the support and respect of every designation of the profession.

### No. 31. January 1, 1828.

The first article is the commencement of an hospital report from Dr. Carter, of the Kent and Canterbury Hospital. Dr. C. informs us that, for several years, intermittents were almost unknown in his neighbourhood—even in Faversham and the marshy lands of its vicinity. The foul fiend was supposed to be extinct—or to have taken his departure for Walcheren or Batavia. But no such thing. Within the last two years, ague has returned—prevailed—in many instances been extremely obstinate—and, in some, has laid the foundation for irremediable disease. It is hardly necessary to say that these effects of protracted ague are chiefly felt in the liver and spleen, leading ulti-

* It has vacillated between aristocracy and democracy—it has been on the verge of free-thinking—and it has been downright methodistical.

mately to dropsy. There is nothing new, in the remedial way, brought forward by Dr. Carter. The quinine and arsenic were, of course, the most effectual remedies for stopping the paroxysms—but every relapse deteriorates the organs and their functions, rendering it necessary to give aperients, mercurials, bitters, and vegetable tonics. We learn from Dr. C. that his colleague, Dr. Chisholm, has successfully practised the plan of Dr. Mackintosh—bleeding in the cold stage. Three fatal cases of organic disease of the liver are detailed; but they present only the usual phenomena of that dire affection.

2. *Phlegmonous Erysipelas.* Mr. Evan Daniel has published some observations on the treatment of the above species of erysipelas—"tending to shew the utility of abstracting blood locally—against which practice, most writers *on this disease* appear to be very much prejudiced." Mr. D. we imagine, labours under a mistake. No modern writers or practitioners are prejudiced against local bleeding in *phlegmonous* erysipelas. It is in the treatment of the superficial or cutaneous erysipelas, that there is considerable discrepancy of opinion. All parties are agreed as to the utility of local depletion in the phlegmonous form—but each has his favourite mode of proceeding. Mr. Lawrence makes a long cut, (he is a cutting kind of chap at all times)—Mr. Hutchison makes short cuts—Mr. Travers cuts both plans, and leaves the operation to leeches—Dr. Babbington and his Greenwich friends make small cuts or punctures with the point of a lancet. These different parties have different views of the modus sanandi of local depletion. The long cutters believe that it is of as great importance to take off tension, as to empty the vessels of the part—the leechers, lancers, and short cutters evidently look to simple depletion as the main benefit to be derived from the measure. In this class Mr. Daniel ranks himself—and this is the whole of the business.

---

3. THE LONDON MEDICAL GAZETTE.

After a considerable note of preparation, and with an address penned (if report be true) by no less a personage than the POET LAUREATE, the MEDICAL GAZETTE has come forth, to be conducted on the "ideal model" of "JUDGMENT, KNOWLEDGE, and GOOD FEELING,"—in short, to be the strenuous advocate of philosophic views and liberal sentiments. For these good intentions we give our cotemporary full credit; and, as far as it is concerned in the laudable endeavour to counteract the baleful influence of the ORGAN of defamation, it will find in us a zealous, though a very humble auxiliary. As there appears to be a greater leaning, however, in the MEDICAL GAZETTE, towards "things that be," than comports precisely with our sentiments, so, it is not impossible that we may have some little collisions in MEDICAL POLITICS. In our differences, however, with the MEDICAL GAZETTE, we hope to conduct ourselves with proper attention to that "ideal model," which our cotemporary has judiciously placed before its eyes, for constant imitation. The Lancet has now got its match in the field. For some time we feared the MEDICAL GAZETTE would wrap itself too much up in its own dignity, and thereby prove tame. It seems to have taken the hint thrown out in our last number. It is now shewing spirit, and we predict that the reign of the Lancet is over! The Hospital Reports, constituting the chief value of all weekly publications, are given in the Medical Gazette infinitely better than in the Lancet, with the incalculable advantage of being TRUE.

MED. GAZETTE, Nos. 4 and 5.

CLINICAL LECTURES.

The first article in the 4th number of our new cotemporary is the substance of a clinical lecture by Mr. Charles Bell, on diseases and accidents of the hip-joint. We may take this opportunity of expressing our opinion, that clinical lectures are the only proper lectures for reporting in a medical Journal. They are, like the debates in a medical society, mere verbal comments on a given subject, which would be lost, if not recorded, and which may be serviceable if noted faithfully and published correctly. Not so with elementary lectures that are constructed for the purpose of annual delivery. These are as much a man's private property as the coat on his back, or the watch in his fob:—and whoever publishes without licence such lectures, is guilty of literary spoliation, according to the laws of honour and conscience, however incognizable such an act may be by the laws of the land. For the truth of this sentiment, we appeal to the breast of every unprejudiced man in the profession.

But, to our business. Mr. Bell, after referring to five or six cases in the wards of the Middlesex Hospital, presenting various specimens of hip-disease—some with the leg of the affected side longer than the other—some with it shorter—some appearing to be on the point of anchylosis—and two in a state which rendered it doubtful whether there was fracture or dislocation of the femur—proceeded to make some general observations on hip-diseases.

This affection is common to infancy as well as age. Mr. B. illustrates its occurrence in the former state, as follows:—an infant was suddenly seized with convulsions, and the fits returned again and again, so that the child was considered to be in great danger. Dr. Denman thought it was owing to dentition, and requested Mr. B. to lance the gums deeply and completely round the jaw. He did so, but no benefit resulted. On careful examination of the naked infant, the disease was found to be in the hip, which was much swelled, tender, and painful on motion. The disease went on, and suppuration surrounded the whole joint. Punctures were often made, and, after a long illness, the little patient finally recovered, though he is lame, from wasting of the head of the femur.

There is another way in which the disease may present itself. A boy at school is observed to limp, and, in the evening, complains of pain and stiffness. He returns to his exercises, and when warmed by these, his lameness and stiffness disappear. Still in the evenings he complains—and, after a time, he is observed to hobble more in his gait. He will now probably acknowledge that he suffers a good deal in the limb—and now it is that medical advice is sought. The examination is to be conducted in the following manner:—

"The first thing you are to do is to make him walk before you, and observe the direction of the toe. You are then to strip him, and place him with the buttocks directly before you. You will observe that on the affected side there is a fulness and a greater breadth betwixt the fissure of the nates and the trochanter major than on the other side; and if the trochanter be firmly pressed inward against the acetabulum, pain will be felt. The patient may in the next place be made to stand erect on both his legs; you then ask him to rest upon the sound limb only, and throw out the other as in abduction. This you will probably find he cannot do without great pain; because, during this movement of the limb, the muscles press the hip-joint, and the head of the bone jars against the delicate and inflamed structure of the acetabulum. If he be now laid straight upon his back on the carpet, you will find an inequality in the length of his limbs. If the patient and others of the family exhibit the peculiar signs of a strumous diathesis, then there is every probability of this being the commencing stage of that very dangerous disease, which is called by authors *Morbus Coxarius*."

Another boy returns from school, and is observed by his mother to be lame or awkward in his gait. He is not conscious of it, and affirms that he played a match of cricket yesterday. On examination, the surgeon perceives that the boy walks lame—yet he can throw the leg about in every direction, without pain. On laying him prostrate on the floor, one leg appears shorter than the other. The short leg will also be found smaller than the other. This depends on scrofula. At a certain period, the boy's leg stops growing, a frequent occurrence during dentition, when the bowels are much disordered. This affection is liable to be confounded with disease of the hip, but it is totally different. Leeches, blisters, and the usual means, are here sometimes applied—with no good, but, probably, bad effects.

In certain cases, (one of which was then in the wards,) the child complains solely of the knee, a common occurrence in disease of the hip-joint, owing, Mr. B. thinks, to inflammation having spread to the obturator nerve, as it passes through the thyroid foramen. The pain is felt in the branches of the nerve, according to a general neurological law. In such cases, when pressure and kneading of the knee occasion no pain, we may suspect the hip-joint to be the seat of the disease.

In respect to shortening of the limb, Mr. B. makes many excellent observations, and we have quoted a passage from this part of his lecture, when speaking of a paper on supposed fractures of the cervix femoris, within the capsule, by Mr. Langstaff. When the disease commences within the joint, it extends its influence to the surrounding parts, which become inflamed, and abscesses form. The patient lies with the body inclined forward, and the knee raised towards the belly. In this way he twists the spine, and inclines the pelvis, drawing it obliquely upwards on the affected side. This apparent shortening of the limb is sometimes erroneously attributed to absorption of the neck of the bone. In the case before him, the limb, on the diseased side, was longer than the other—yet still it was owing to twisting of the pelvis. But the apparent shortening of the limb is the common occurrence, and if this be perceived on the second or third day after the commencement of acute inflammation of the hip, the surgeon may begin to fear that he had made a mistake—that there had been a fracture or dislocation of the femur that had not been detected.

"In order to satisfy yourselves whether there is real, or only apparent shortening of the limb, make the patient lie even upon his back, in as straight a position as he can, and then search for the superior spinous processes of the ilium on both sides. By drawing a tape between these two points, it will be seen whether the pelvis is situated obliquely or not. If the pelvis be placed exactly in a straight position, then the difference in the length of the two limbs no longer exists : the heels may be made to meet and correspond exactly. But whenever the examination is over, the patient gradually resumes his former position of ease, and then you find that the limb is drawn upwards, and appears shortened just as before."

A case in elucidation is given, and also a wood-cut, shewing the alteration in the head and neck of the femur produced by inflammation. This clinical lecture is creditable to Mr. Bell, and affords him opportunities of shewing his ingenuity in the explanation of morbid phenomena connected with the mechanism of the human frame.

---

### 4. THE MEDICAL AND PHYSICAL JOURNAL.

This Journal originated in cow-pox, in the last year of the last century. Its success was far beyond that of any other medical journal that had previously appeared. It became nearly as epidemic as vaccination. We have seen documents, in the possession of the late Mr. Thorne, the original printer of the journal, proving that its circulation, for some years, amounted to nearly 3000 copies per month! It will soon have attained its 60th volume —a grand climacteric in periodical literature! A list of its editors would nearly cover a page of our work—and the various tints and hues which the Medical and Physical Journal received from its successive directors, are far beyond our power of painting. We wish it every success.

The Number for Jan. (19, New Series) opens with a paper on the physiology of the circulation, by Mr. James, of Exeter; in which nothing new is advanced, and nothing old is established or refuted. Mr. James seems to come to the conclusion, (to which we long ago came, and which we have repeatedly published,) that the blood does not go along the arteries in waves, but that, at each ventricular contraction, "a shock is diffused over the whole arterial system, which propels the blood in a longitudinal direction." There is, no doubt, a very trifling instantaneous dilatation of the whole tree of arteries at the same time, the recoil of which feeds the capillary system till the next ejection of blood from the heart. Mr. James affects to treat the suction-influence of the chest and heart as chimeras of the imagination. It is needless to say, that he offers nothing like facts to substantiate this negation.

In the second article, Sir George Gibbes continues his speculations on life, on animalcula, on monades, on ultimate living particles, terminating rudiments, &c. through which speculations we dare not follow him. Sir George appears to be enamoured of the following "very novel and most curious" doctrine.

"All the tissues of the animal body are shewn to be ultimately resolvable into minute globules, and that these globules, as they are successively disengaged from the mass, exhibit distinctly a power of spontaneous activity, moving about rapidly in all directions. In short, they become animalcula, possessing the power of locomotion, and have been named monades. It appears that these bodies are capable of existing as animals or vegetables, and of forming elementary parts of either. Thus, according to the above view of the subject, we arrive at the singular conclusion, that the human body, with all its organs, is built up of animalcula, and that it is a congeries of countless millions of organized beings, each capable of living in a separate state, and, perhaps, exercising some of the functions of individual life, whilst incorporated with our system. It is not certain, but it is at least probable, that these monades form the last link in the chain of organic life, and that, beyond them, there is nothing but the ultimate gazeous elements."

The author of this curious theory believes the process of digestion to consist merely in the operations necessary to separate these monades from the combibations in which they existed in the animal and vegetable substances that form our food, while assimilation is the mere transport of the monades to the various parts of the body.

How will Sir Gilbert Blane, Professor Brande, and Doctor Reece chuckle, when they peruse this theory, so beautifully corroborative of their doctrine, that Thames water is both wholesome and *fattening!* No wonder that it is so, when it contains so many millions of animalcula, all ready disengaged to fly to the different parts of the body, without any digestive process being necessary!

*Vaccination of the Sultan's Children.* Dr. Baron, of Gloucester, has transmitted the important information, that three of the Grand Seigneur's children have undergone vaccination. It appears that His Turkish Majesty has been inoculated with the contagion of European discipline, ever since the destruction of the Janissaries. Nothing is now heard, in Constantinople, but military music—nothing played except European airs—nothing seen but drum-majors, great canes, muskets and bayonets—and the Grand Seigneur himself, in a General's uniform, ordering manœuvres! This fine flourish introduces us to the important intelligence, that Dr. Auban has had the honour of vaccinating three of the Sultan's children, in order to complete the revolution that has been effected among the followers of the Prophet.

*Paraplegia.* Dr. Thomas, of Devonport, has detailed a case of this kind, which occurred in the person of a sailor, aged 43 years. He had suffered an attack of fever fifteen years previously in India, and one of acute rheumatism in the Winter of 1824. After sleeping in a damp room last Spring, he felt pain in the left side, extending to the back. This was followed by weakness and numbness in the lower extremities, constipation of bowels, dysury, soon amounting to retention. There was no affection of the head. We need not detail the treatment. For a time the patient improved—but afterwards he became completely hemiplegiac. Ulceration took place over the sacrum, and then the paralysis of the lower extremities diminished—till it might be said to be almost gone. Ulceration of the bladder, however, occurred, and of this the patient died. No dissection could be obtained. The head remained free from disorder all the time.

Dr. Thomas seems to have brought forward this case, with the view of assisting Dr. Burder in refuting the doctrine of the late Dr. Baillie, that paraplegia generally depends on affection of the brain. But there is hardly any one who maintains such a doctrine now—and the case of Dr. Thomas is defective, from want of dissection. We have no doubt, however, that, in the above case, the disease was in the spinal marrow, and that the ulcer over the sacrum acted the salutary part of a caustic issue.

The hospital reports, in the Medical and Physical Journal we shall amalgamate with others under their proper heads.

---

### 5. LANCET. JANUARY 5, 1828.

It is not our intention, except on very extraordinary occasions, to notice the *elementary* lectures in the columns of the Lancet. They are calculated for two classes of readers—those who have lost their dentes sapientiæ from old age—and those striplings who have not yet cut them. The lectures, indeed, of a Cooper, an Abernethy, and a few others, *did* possess interest—but now—we have had satis superque. The *clinical* lectures, as we have elsewhere observed, are the proper subjects for reporting.

In this Number are given some clinical remarks, by Dr. Elliotson, on chronic affections of the brain. The subjects in the hospital were chiefly males, between the age of 25 and 40—the causes, in many instances, were external injuries. The symptoms were, generally, pain in the head, drowsiness, vertigo, throbbing, dilated pupils, tinnitus aurium, &c.—in short, the symptoms of chronic inflammation—and the terminations were too often in hemiplegia, epilepsy, convulsions, &c. The post-mortem appearances, in Dr. E.'s practice, were those which others have observed—effusion, thickening of membranes, ramollissement, &c. The treatment may be very easily anticipated,—spare diet,—open bowels—cold, repeated blisters, cupping and leeching ("almost endlessly") to the head—abstraction of blood from the hypochondria or epigastrium—mercury, which should never be omitted. A case was related, and a brain exhibited where there was serous fluid found on the surface of the brain, as well as in the ventricles—also a quantity of limpid fluid on the medulla spinalis. The reporter makes Dr. Elliotson to say that the case in question is one not only instructive in itself but as "*mutilating*" against the opinion advanced by Majendie, that the fluid found in the ventricles comes from the spine entirely, or in the greater part; and, consequently, that our remedial measures should be chiefly directed to that region. We do not deem it necessary to enter into the investigation here, whether or not there is a communication between the ventricles of the brain and the spinal canal.

### COOPER *versus* LAWRENCE.

Cantare pares, et recantare parati.

There is, at this moment, a tremendous conflict between two surgical instruments—the trephine and the scalpel—the Lancet being, of course, the bottleholder. Mr. Lawrence appears to have thought that there would not be sufficient space on Mr. Cooper's head for a 14 inch incision—and therefore he has made several perforations to get at the spinous artery of his antagonist. Mr. Cooper, on the other hand, has taken up the scalpel, and, avoiding all attempts to get at the *dura* mater of Mr. Lawrence, amuses himself by making short, but pretty numerous incisions into the *soft* parts of the enemy. How this deadly contest will end we cannot yet predict. Both parties appear at home in the use of their weapons, and, it is to be feared, that blood will be spilt and bones broken before the victory is proclaimed.

### DUEL.

We have now to record a much more serious kind of fighting than that with the scalpel and trephine. A duel was fought, on Saturday week, between Dr. Forbes, of Argyll-street, and Mr. Thompson, a young surgeon, a pupil at the Eye-Infirmary, to which Dr. Forbes is physician. No blood was shed, but a ball went through Mr. Thompson's hat, when the seconds interfered. The circumstances that led to this meeting are pretty well known. Very late in the evening, before Mr. Guthrie's action was to have come on against the Lancet, Dr. Forbes wrote to Mr. Guthrie, intimating that, if interrogated in court, he would be forced to condemn certain parts of Mr. Guthrie's practice, or words to that effect. This, of course, stopped proceedings in court next day, and the business, we suppose, is ended for ever. It seems that Mr. Thompson was indignant on this occasion, and insulted Dr. Forbes—hence the duel. We join with our cotemporary in considering the above as a striking illustration of the tumultuous state of our profession, "It is one among the daily proofs of the incalculable mischief resulting from that system of depravity in the medical press, which has thus literally set man in hostility to man, for the profit of a ——— ———." We dare not close the sentence from our cotemporary, lest we should have another judicial action on our hands! We confidently believe that the "system of depravity" is beginning to totter—and we hope to give it a shove before the end of the year 1828.

